# Simultaneous Estimation of Esomeprazole and Domperidone by UV Spectrophotometric Method

**DOI:** 10.4103/0250-474X.40351

**Published:** 2008

**Authors:** S. Lakshmana Prabu, A. Shirwaikar, Annie Shirwaikar, C. Dinesh Kumar, A. Joseph, R. Kumar

**Affiliations:** Department of Pharmaceutical Quality Assurance, Manipal College of Pharmaceutical Sciences, Manipal - 576 104, India; 1 Department of Pharmaceutics, Manipal College of Pharmaceutical Sciences, Manipal - 576 104, India; 2 Department of Pharmacognosy, Manipal College of Pharmaceutical Sciences, Manipal - 576 104, India; 3 Department of Pharmaceutical Chemistry, Manipal College of Pharmaceutical Sciences, Manipal - 576 104, India

**Keywords:** Esomeprazole, domeperidone, λ max, spectrophotometric method

## Abstract

A novel, simple, sensitive and rapid spectrophotometric method has been developed for simultaneous estimation of esomeprazole and domperidone. The method involved solving simultaneous equations based on measurement of absorbance at two wavelengths, 301 nm and 284 nm, λ max of esomeprazole and domperidone respectively. Beer's law was obeyed in the concentration range of 5-20 μg/ml and 8-30 μg/ml for esomeprazole and domperidone respectively. The method was found to be precise, accurate, and specific. The proposed method was successfully applied to estimation of esomeprazole and domperidone in combined solid dosage form.

Esomeprazole magnesium trihydrate^1^ (ESO) is chemically bis(5-methoxy-2-[(S)-[(4-methoxy-3,5-dimethyl-2-pyridinyl)methyl]sulfinyl]-1-H-benzimidazole-1-yl) magnesium trihydrate, a compound that inhibits gastric acid secretion. Esomeprazole is cost effective in the treatment of gastric oesophageal reflux diseases. Esomeprazole is the S-isomer of omeprazole, the first single optical isomer proton pump inhibitor, generally provides better acid control than current racemic proton pump inhibitors and has a favorable pharmacokinetic profile relative to omeprazole^2^. Domperidone^3^ (DOM) chemically, [5-chloro-1-[1,3-(2,3-dihydro-2-oxo-1H-benzmidazole-1yl)propyl)-4-piperdinyl-1,3-dihydro-2H-benzimidazole-2-one] is a dopamine antagonist. A detailed survey of literature revealed the estimation of omeprazole by gas chromatographic method^4^, UV spectrophotometric method^5^–^6^, TLC^7^ and several HPLC^8^–^20^ methods. Estimation of DOM included spectrophotometric methods^21^–^22^, HPLC^23^–^26^ and HPTLC^27^ in dosage forms. Combination of these two is used for the treatment of gastric esophagus reflux disease. However, no references have been found for simultaneous determination of ESO and DOM in pharmaceutical formulations. A successful attempt has been made to estimate two drugs simultaneously by spectrophotometric analysis.

A Shimadzu UV/Vis spectrophotometer, model 1601 (Japan) was employed with spectral bandwidth of 0.1 nm and wavelength accuracy of ± 0.5 nm with automatic wavelength correction with a pair of 3 mm quartz cells. ESO, DOM (obtained as gift samples from Themis Laboratories Pvt. Ltd., Thane), methanol (Merck India Ltd., Mumbai) and distilled water were used in the present study.

Stock solutions (500 μg/ml) of ESO and DOM were prepared by dissolving separately 50 mg in 50 ml of methanol in 100 ml volumetric flasks, and the volume was made up to 100 ml with distilled water. The maximum absorbances of ESO and DOM were obtained at 301 nm (λ_1_) and 284 nm (λ_2_), respectively. ESO and DOM showed linearity with absorbance in the range of 5-20 μg/ml and 8-30 μg/ml at their respective maxima, which were validated by least square method. Coefficients of correlation were found to be 0.9972 for ESO and 0.9986 for DOM. For simultaneous estimation of ESO and DOM, a series of standard solutions in concentration range of 5 to 20 μg/ml, were prepared by diluting appropriate volumes of the standard stock solutions. The scanning solutions of ESO and DOM were carried out in the range of 200 to 400 nm against water as blank for obtaining the overlain spectra that are used in the analysis (fig. 1). Absorbance and absorptivities of series of standard solutions were recorded at selected wavelengths λ_1_ and λ_2_.

**Fig. 1:**
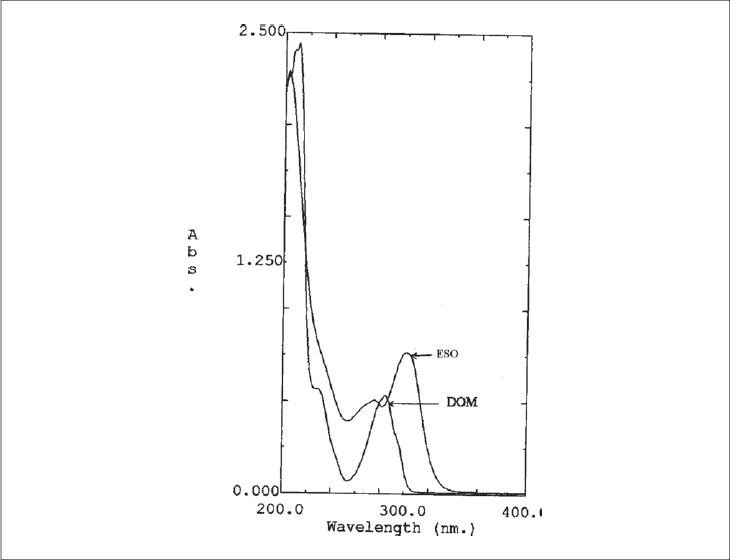
Overlain spectra of ESO and DOM. Overlain spectra of esomeprazole (ESO) and Domperidone (DOM) in water.

The absorptivity values for ESO and DOM at 284 nm were 243 ± 1.73 and 266 ± 1.80, respectively. At 301 nm the absorptivity of ESO and DOM were 383 ± 1.87 and 47 ± 1.73, respectively. The optical characteristics and regression values for the calibration curve are presented in Table 1. The method employed simultaneous equations using Cramer's rule and matrices (C_1_ = λ_2_ε_2_ × Aλ_1_−λ_1_ε_2_ × Aλ_2_/λ_1_ε_1_ × λ_2_ε_2_−λ_1_ε_2_ × λ_2_ε_1_ and C_2_ = λ_1_ε_1_ × Aλ_2_ − λ_2_ε_1_ × Aλ_1_ / λ_1_ε_1_ × λ_2_ε_2_ − λ_1_ε_2_ × λ_2_ε_1_). A set of two simultaneous equations was framed using the mean of absorptivity values, given as Aλ_1_ = 243C_1_ + 266C_2_ and Aλ_2_ = 383C_1_ + 47C_2_, where, C_1_ and C_2_ are the concentrations of ESO and DOM respectively in simple solution (μg/ml). Aλ_1_ and Aλ_2_ are the absorbances of the sample solutions measured at 284 and 301 nm, respectively.

**TABLE 1:** REGRESSION AND OPTICAL CHARACTERISTICS OF ESOMEPRAZOLE AND DOMPERIDONE

Parameters	ESO	DOM
Beer's law range	5-20 μg/ml	8-30 μg/ml
Molar absorptivity (0.001 absorbance unit/mole.cm/dm^3^)	1.864 × 10^4^	1.133 × 10^4^
Sandell's sensitivity (μg/cm^2^/0.001 absorbance unit)	0.0412	0.0376
Regression coefficient (r)	0.9972	0.9986
Slope	0.2038	0.1285
Intercept	0.1729	0.1505

Twenty capsules were weighed accurately. The average weight was determined and then ground to a fine powder. A quantity equivalent to 30 mg of DOM and 20 mg of ESO were transferred to a 100 ml volumetric flask. The contents were ultrasonicated for 10 min with 50 ml of methanol, made to volume with distilled water. Then the solution was filtered through a Whatman filter paper (No. 40). The filtrate was further diluted with distilled water. The absorbance of the resulting solution was measured at 284 and 301 nm. The result of the analysis of the capsule formulation is presented in Table 2.

**TABLE 2:** ANALYSIS OF COMMERCIAL FORMULATION

Brand A		Brand B	
	
% ESO	% DOM	% ESO	% DOM
99.54 ± 0.89	100.21 ± 0.63	99.92 ± 1.05	99.65 ± 0.77

Brand A: Ignis 20 (Alembic Ltd, Vadodara) and Brand B: Izra - D (Unichem India Ltd, Mumbai) containing 20 mg of Esomeprazole magnesium trihydrate equivalent to 20 mg of esomeprazole and 30 mg of domperidone

To study accuracy, reproducibility and precision of the proposed methods, recovery studies were carried out at three different levels by addition of standard drug solution to preanalysed sample. Results of recovery studies were found to be satisfactory and the results are presented in Table 3.

**TABLE 3:** RECOVERY STUDIES

Drug in standard mixture solution (μg/ml)		% Recovery ± SD		Coefficient of variance	
		
ESO	DOM	ESO	DOM	ESO	DOM
2	2	99.36 ± 0.144	99.30 ± 0.261	0.332	0.511
4	4	99.32 ± 0.271	99.11 ± 0.447	0.424	0.358
6	6	99.64 ± 0.355	99.52 ± 0.591	0.297	0.493

SD stands for standard deviation, the results are mean of three readings (*n* = 3)

The proposed method for simultaneous estimation of ESO and DOM in combined sample solutions was found to be simple, accurate and reproducible. Beer's law was obeyed in the concentration range of 5-20 μg/ml and 8-30 μg/ml for esomeprazole and domperidone respectively. Co-efficient of variation was found to be 0.9972 and 0.9986 for ESO and DOM, respectively. The percentage recoveries were found to be in the range of 99.32 to 99.64% and 99.11 to 99.42% for ESO and DOM, respectively. Once the equations are determined, analysis requires only the measuring of the absorbance of the sample solution at two wavelengths selected, followed by a few simple calculations. It is a new and novel method and can be employed for routine analysis in quality control laboratories.
